# "Sequencing-grade" screening for *BRCA1 *variants by oligo-arrays

**DOI:** 10.1186/1479-5876-6-64

**Published:** 2008-10-30

**Authors:** Alessandro Monaco, Filippo Menolascina, Yingdong Zhao, Stefania Tommasi, Marianna Sabatino, Ross Fasano, Angelo Paradiso, Francesco M Marincola, Ena Wang

**Affiliations:** 1 Department of Transfusion Medicine, Clinical Center, National Institutes of Health, Bethesda, MD, USA; 2 Department of Bioinformatics, University of Bari, Italy; 3 Biometrics Research Branch, National Cancer Institute, National Institutes of Health, Bethesda, MD, Italy; 4 Clinical Experimental Oncology Laboratory, Istituto Tumori IRCCS "Giovanni Paolo II", Bari, Italy

## Abstract

The need for fast, efficient, and less costly means to screen genetic variants associated with disease predisposition led us to develop an oligo-nucleotide array-based process for gene-specific single nucleotide polymorphism (SNP) genotyping. This cost-effective, high-throughput strategy has high sensitivity and the same degree of accuracy as direct sequencing, the current gold standard for genetic screening. We used the *BRCA1 *breast and ovarian cancer predisposing gene model for the validation of the accuracy and efficiency of our strategy. This process could detect point mutations, insertions or deletions of any length, of known and unknown variants even in heterozygous conditions without affecting sensitivity and specificity. The system could be applied to other disorders and can also be custom-designed to include a number of genes related to specific clinical conditions. This system is particularly useful for the screening of long genomic regions with relatively infrequent but clinically relevant variants, while drastically cutting time and costs in comparison to high-throughput sequencing.

## Background

High throughput $1,000 whole genome sequencing may be rapidly approaching[1,2], meanwhile, a clinical need exists for the screening of genes whose polymorphisms determine disease predisposition, natural history or therapeutic outcome. Screening of the *BRCA1 *(OMIM 113705) cancer predisposition genes is an example of such a situation and it was well exemplified by [3,4] by Gerhardus *et al *[5], who systematically reviewed 3816 publications to estimate the accuracy of diagnostic methods used for the detection of *BRCA1 *and *BRCA2 *mutations. They concluded that many of the alternative screening methods were as time- and cost-intensive as direct sequencing, but did not provide the same definitive information. In addition, many of these methods could not be recommended for routine screening because of low sensitivity. Denaturing high-performance liquid chromatography was shown to outperform other methods but still required to be complemented by sequencing. Significantly, none of the techniques evaluated in the study, including direct sequencing, could detect large rearrangements, such as whole exon germline deletions/insertions.

**Figure 1:**
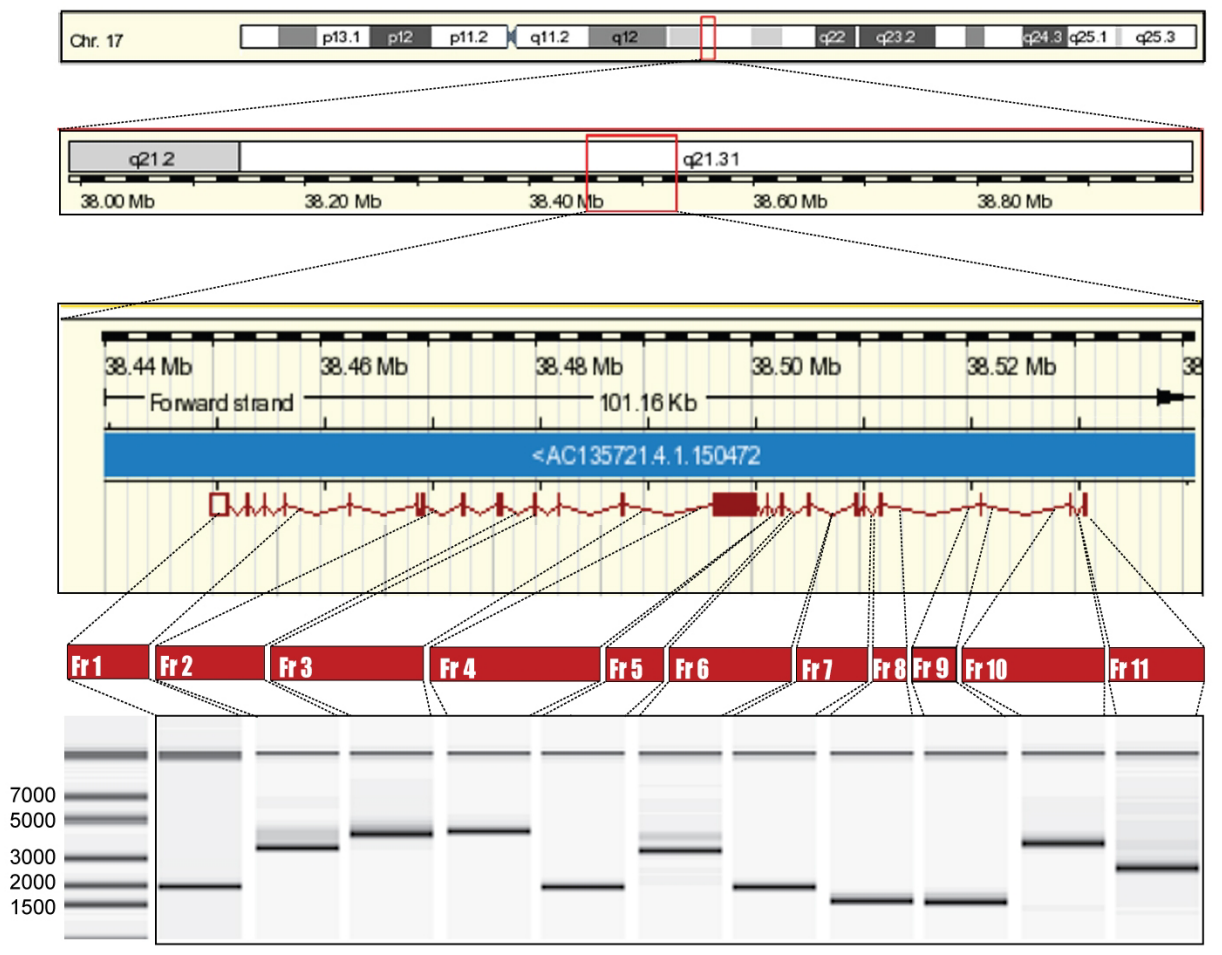
**Chromosomal location and genomic mapping of the *BRCA1 *locus and sub-fragments amplified for genomic analysis.** The correct size for each amplicon is shown in the lower panel.

Germline mutations in *BRCA1 *account for a small but significant proportion of breast cancers. Genetic testing has been routinely applied to women from high risk families since 1994 [6,7]. *BRCA1 *spans an approximately 81 Kb region encompassing 24 exons (22 coding), and so any screening method must confront the challenge of monitoring this large genomic region over which the relevant variants are scattered[8] (Figure 1). Sequencing using semi high-throughput Sanger sequencing technology remains the gold standard for evaluating the *BRCA1 *gene despite its relatively high cost and time commitment[5].

## Results and discussion

We used a previously described flourimetric SNP detection strategy based on the proportional hybridization of test and reference material with an oligonucleotide array platform [9] to design a *BRCA1*-specific array covering the entire coding region. This array was capable of detecting SNPs and/or gene rearrangements (insertions and deletions), even in heterozygous conditions. At reasonable cost, we used sequence-specific probes to query hundreds of kilobases within a single reaction. The array design included 1,423 consensus oligo probes arranged at 4-nuclotides tiling based on arbitrarily selected wild type *BRCA1 *reference sequence [9] to cover all the exonic regions of *BRCA1 *and part of the intronic regions (Table 1). Oligo probes were designed in variable size (from 18 nucleotide to 25) to maintain constant the melting temperature [10-12]. In addition, 38 exonic and 31 intronic oligo-probes representing known variants of *BRCA1 *were designed according to Ensembl SNP database  where the variant SNP was placed in the centermost position of the probe to enhance the specificity and discriminative power of the hybridization[9]. An arbitrarily-selected wild-type sequence was derived from Ensembl database . Reference sample consisted of genomic DNA extracted from MCF-10A, a mammary epithelial cell line previously shown by sequencing to be homozygous at the *BRCA1 *locus. The sequence of the *BRCA1 *gene in MCF-10A was not completely identical to the wild-type consensus sequence but represented the closest available match.

**Table 1:** Estimated cost and time requirements for typing of the BRCA1 gene by direct sequencing vs SNP array

	***Consumables supplies***	***Equipment***	***Personnel***	***cost/react***	***Total Cost/sample BRCA1 gene (35 fragment)***	***Time***	***Time/20 samples***
**Direct sequencing**	$11.30	$7.30	$10.08	$28.68	$1,003.80	approx 2 working days	approx 20 working days

**SNP array**	$38.74	$12.50	$8.30	$59.54	$59.54	less than 3 working days	less than 3 working days

**Figure 2:**
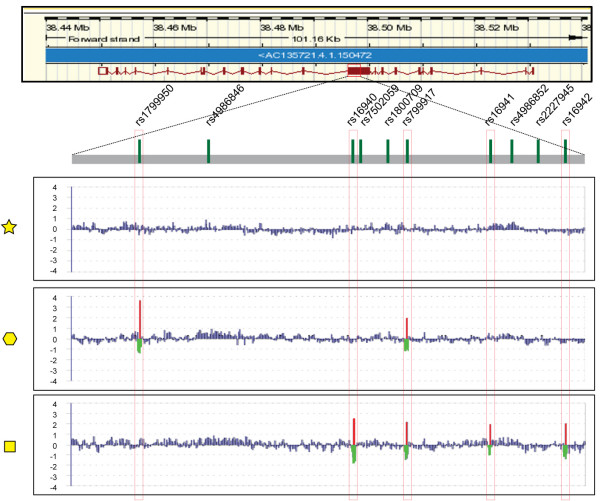
**Representative example of the graphical representation of SNPs for Fragment 4 in three patients' samples.** The yellow symbols (Star, Hexagon, Square) relate to cases shown in Figure 3A.

Probes with 3'amine modification were spotted onto a 3D-link-activated array slide by covalent immobilization (GE Healthcare) using OmniGrid robotic printer (GeneMachine). Genomic DNA was extracted using Qiagen blood extraction kit. PCR amplification was performed using Phusion polymerase (F-530L, Finnzymes) according to company instructions. Eleven primer sets were used to amplify the entire coding region and parts of the intronic regions (Figure 1). A T7 promoter sequence was attached to the 5' end of each forward primer to allow subsequent *in vitro *transcription. After denaturing at 98°C for 30 sec, PCR reactions were cycled 30 times at 98°C for 7 sec, 68°C for 20 sec, 72°C for 2 min followed by 72°C for 2' 30". The PCR amplicon size was confirmed using an Agilent 2100 bioanalyzer (Figure 1, bottom panel). Three microliters of each amplicon from the same patient were combined together and purified with a Microcon YM-100 spin column (Millipore, Bedford, MA) to remove primers. Eight microliters of the total volume (30 ul) of the purified PCR *BRCA1 *amplicon mixture from each patient was subjected to *in vitro *transcription using T7 Megascript kit (Ambion). The reaction was run for 8 hours at 30°C. Isolation of amplified RNA (aRNA) was performed by TRIZOL purification. Three micrograms of purified aRNA were fluorescently-labeled with a reverse-transcription reaction in the presence of 2 μg of random hexamer, 5 μl 4× first-strand buffer, 2 μl 0.1 M DTT, 1 μl RNasin, 2 μl of 5 mM low T dNTP, 2 μl of 2 mM Cy3 (reference sample) or Cy5 (test sample) dUTP (Amersham, Piscataway, NJ) and 2 μl of SSII (Invitrogen). Labeled cDNA were purified and co-hybridized on to *BRCA1 *chip in the presence of blocking reagents after denaturing. Hybridized arrays were scanned at 10 μm resolution on a GenePix 4000 scanner (Axon Instruments, Inc., Foster City, CA) at variable PMT voltage to obtain maximal signal intensities with <1% probe saturation. Resulting tiff-format images were analyzed to calculate fluorescence intensities and log2 Ratio values, which were normalized and portrayed graphically[9] (Figure 2).

**Figure 3:**
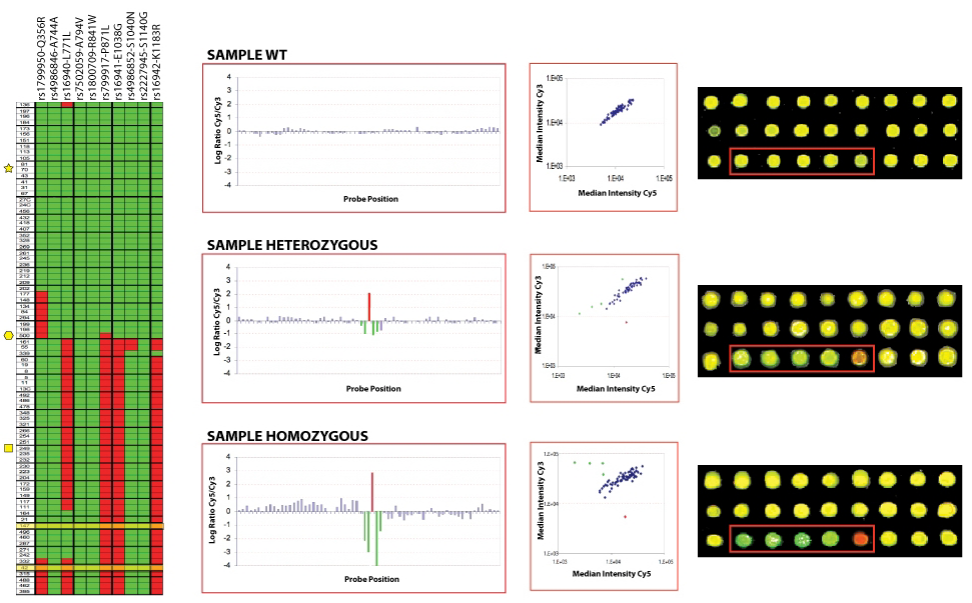
**(A) – Heat map summarizing results for fragment 4 from 85 patients with breast cancer tested for *BRCA1 *mutation at the Bari National Cancer Institute.** In red are identified SNPs which are annotated at the top of each column. Each row represents a patient's sample. The two cases highlighted in yellow refer to two patients whose array-based analysis could not be confirmed by sequencing due to insufficient DNA. Cases are self organized using Eisen's cluster program according to individual proximity to each other (Pearson's correlation). The yellow symbols (Star, Hexagon, Square) recall the cases shown in Figure 2. (B) Blow up of a graphical representation in fragment 4 of balanced hybridization between identical test and reference samples (top panel), a heterozygous (middle panel) and a homozygous (bottom panel) difference. SNPs in the test sample are shown as gain of signal in red while loss of signal in the consensus wild type signal is reflected by the four green probes. To the side is the region is represented as a scatter plot and as an actual image from the array.

A specific pattern can be seen which denotes the presence of a SNP. The feature of this pattern is characterized by the red signal deflection (Cy5) representing the specific hybridization of the test sample to the oligo-probe for the specific SNP and a flanking region green signal (Cy3) deflection including overlapping wild type consensus oligo-probes for each side around the SNP. Homozygous samples will hybridize more strongly and have higher red and green fluorescent intensity as compared to heterozygous samples (see also Figure 3B). In addition, the presence of green deflections (Cy3) in consecutive probes flanking the region of a putative unknown variant would indicate the presence of a novel SNP if no corresponding red spike (Cy5 SNP-specific probe) could be detected in that region to indicate a known specific variant.

To evaluate the sensitivity and specificity of the process, we compared results obtained with the oligoarray against those from direct sequencing. As part of an ongoing clinical protocol, samples for *BRCA1 *and *BRCA2 *mutational analysis were obtained from 85 consecutive patients with familial breast and/or ovarian cancer[13]. Patients were seen and signed informed consent at the Genetic Counselling Program, Clinical Oncology Laboratory, at the Bari National Cancer Institute (DNV Certificate N. CERT-17885-2006-AQ-BRI-SINCERT). Only patients classified as having a higher than 10% probability of carrying a *BRCA1 *or *BRCA2 *mutation were enrolled. This risk was calculated using the New Myriad II program, which references an individual's TNM classification U.I.C.C., cyto-histological differentiation grade, estrogen receptor (ER) and progesterone receptor (PgR) status, tumor content and whether there is a history of breast or ovarian cancer among relatives.

Fragment 4 of the *BRCA1 *locus contains several SNPs associated with the predisposition for developing breast and ovarian cancer and was used for SNP analysis and validation by direct sequencing (Figure 2). To demonstrate the principle, data were portrayed for individual fragments (sub-arrays) after fragment-specific normalization to graphically display the presence of SNPs along the sequence (as previously described[9]; Figure 2). Consistent calls identifying SNPs present in the reference sample (that was not completely identical to the wild-type consensus sequence) in all cases were excluded from the analysis because representative of variations in the reference MCF-10A cell line and not related to the test sample. This fragment-specific normalization corrects sequence-specific and amplicon-specific variation in intensity that may cause imbalanced hybridization as tested using sequence identical samples differentially labeled and hybridized on the same chip for calibration purposes (see example in Figure 2, top panel). This normalization does not affect the intra-fragment reference/test ratio measurements.

A custom made software SNPpositioner uses an algorithm that queries the Graphical User Interface to select pre-determined chromosomal regions relevant to the analysis (individual fragments in this case). Probe logRatio were first averaged from duplicated spots followed by the "Local Amplicon-oriented Normalization Algorithm" (LANA). This LANA approach is used to sort individual probes implementing the two nearest flanking probes summarized below:

*T*(log *Ratio*_*i*_) = 2*log *Ratio*_*i *_- log *Ratio*_*i*-1 _- log *Ratio*_*i*+1_

Data analysis, therefore, is performed blindly and automatically to identify variant sequences when the transformed Cy5/Cy3 logRatio [T(logRatio)] of a probe is above and/or below a fragment-defined baseline cutoff value which is two standard deviations in the current settings. This algorithm objectively identifies sequence variations without any subjective manipulation the oligo-array data. The analysis was carried out blind (only the reference was completely sequenced for the *BRCA1 *locus) and it was automated using our custom software that made calls without input from previous sequencing information. Thus, the study was used as a training set for the program.

To ensure the accuracy of this technology and analysis software, the output SNP information was compared with sequence-based analysis of 2 kilobases region in fragment 4 (Figure 3A). This comparison identified complete concordance between SNPs identified by SNPpositioner and those made by sequencing analysis for 83 of the 85 patient samples (highlighted in yellow in Figure 3A; two patient samples could not be sequenced due to insufficient DNA and, therefore, the accuracy of the array could not be tested in those). In these 85 patients, the oligo-array detected 15 non-synonymous, 4 synonymous and 10 intronic SNPs. No novel SNPs were identified in this previously well-characterized Italian population [4,6,13]. In about 50% of patients tested, three SNPs (P871L, K1183R and E1038G) were consistently present, indicating possible haplotype linkage. When cross-referenced with clinical-pathological information, these three linked SNPs identified a cluster of individuals possessing a higher percentage of cyto-histologically differentiated cancers as compared with the other patients (71% [27/38] vs 50% [19/38] of G1-2 tumours; p = 0.05). These patients also had a lower probability of carrying a deleterious *BRCA1 *or *BRCA2 *mutation (74% [31/42] vs 56% [24/43] of cases with Myriad probability ≤ 10%, p = 0.06) [4].

Although the oligo-array's accuracy was only confirmed with sequencing by fragment 4 of the *BRCA1 *locus, it could be expected that the same accuracy would be observed with other fragments. Thus, the whole *BRCA1 *gene can be analyzed with one oligo-array reaction and have the same accuracy as at least 70 sequencing reactions (about 35 kb). In addition, the automated data interpretation eliminated regions of balanced hybridization limiting the analysis to only those few regions flagged by the software to contain SNPs, therefore, greatly simplifying the analysis. A comparative analysis of the time and cost of the two techniques is shown in Table 1. Our estimates of the cost of sequencing for the *BRCA1 *were similar to others' reports [5].

## Conclusion

In summary, the process presented here is an accurate and efficient screening strategy for gene-specific detection of clinically or scientifically relevant genomic variants. This validation should be regarded as a further improvement in the efficiency of genetic testing as discussed by Gerhardus *et al *[5]. Contrary to previously sequencing-on-chip methods [14-18], this method can detect known gene variants [9] with high sensitivity while using a much smaller number of oligos. Indeed, other systems comparable to the present in potential accuracy such as "on chip sequencing" cover a complete gene sequence tiling oligos with a 1 nucleotide overlap and including probes for each possible nucleotide permutation for each base position. This study clearly shows that for practical purposes, such as clinical-grade genetic testing, this extensive approach is not necessary and wasteful; in fact, although in theory it eliminates the requirement for sequencing, in practice it requires a large number of oligos to cover areas that are in most cases non-polymorphic or test genes whose polymorphisms are in most cases known (as the *BRCA1 *gene). Our process can theoretically flag the occurrence of unknown variants based on the sequential signal loss pattern in tiled consensus oligo probes, although not tested in this study in which well-characterized patients were screened; in this case sequencing in the search for new variant sequences could be focused to extremely limited areas in rare patients (only those patients carrying novel SNPs). We estimate that this process could reduce the need for direct sequencing to less than the 1% of present norms. In addition, because of the small number of oligos needed, as compared with sequencing-on-chip technologies, this strategy dramatically reduces the production costs. It may also allow the inclusion of several genes relevant to a specific disease process to be analyzed simultaneously at "sequence-grade" levels using high-density platforms.

## Competing interests

The authors declare that they have no competing interests.

## Authors' contributions

AM performed the optimization of the conditions, co-designed the experiment, run the samples on the chip and sequenced them. He also analyzed the data and compared the results of the two techniques. FM developed the software to analyze the data. YZ validated the software performing tests to evaluate the correct functioning. ST collected the samples and supported the development of the technique. MS co-performed the samples run and contributed to the analysis of the data. RF co-performed the samples run and contributed to the analysis of the data. AP coordinated the project from the samples collection to the output of the data. FMM directed and Co-designed the project, supervised all the phases of the process, contributed to the validation of the technique and the analysis of the data. EW developed the technique, co-designed and supervised all the phases of the project. She also took part in the development and validation of the software and in the analysis of the data.
